# Dynamic symptom networks in etomidate use disorder: a cross-lagged panel network analysis of craving, compulsive drug seeking, and cognitive control

**DOI:** 10.3389/fpubh.2025.1725123

**Published:** 2026-01-20

**Authors:** Juan Le, Ying Tang, Xingmin Wang, Qiuping Huang, Xinxin Chen, Tefu Liu, Zhigang Chen, Yunde Tang, Qian Liu, Lin Zhao, Hongxian Shen, Zhenjiang Liao

**Affiliations:** 1 Department of Psychiatry, National Clinical Research Center for Mental Disorders, The Second Xiangya Hospital of Central South University, Changsha, Hunan, China; 2 Department of Psychology, School of Humanities and Management, Hunan University of Chinese Medicine, Changsha, Hunan, China; 3 Hunan Lituo Compulsory Isolation and Drug Rehabilitation Center, Hunan Lituo Drug Rehabilitation and Recovery Center, Changsha, Hunan, China; 4 Hunan Provincial Lushan Compulsory Isolation Detoxification Center, Changsha, Hunan, China

**Keywords:** cognitive control ability, compulsive drug seeking, cross-lagged panel network analysis, drug craving, etomidate use disorder

## Abstract

**Background:**

Etomidate abuse has emerged as a novel psychoactive-substance threat in China, yet the symptom architecture and temporal dynamics of etomidate use disorder (EUD) remain poorly characterized. This study aims to elucidate the static and dynamic interactions among craving, compulsive drug seeking and cognitive-control deficits in individuals with EUD through cross-sectional and longitudinal network analyses.

**Methods:**

From June 2023 to December 2024, a total of 361 male individuals diagnosed with EUD, with a median age of 21 years (interquartile range: 18–26), were assessed upon admission (T0) and one month thereafter (T1) at a rehabilitation center in Hunan Province. Craving was measured with the Drug Desire Questionnaire; compulsive seeking with the Obsessive Compulsive Drug Use Scale; impulsivity with the Barratt Impulsivity Scale; and cognitive control via the Stop Signal Task. Cross-sectional symptom networks were estimated using Gaussian graphical models with GLASSO regularization. Temporal dynamics were examined with cross-lagged panel network modeling. Network centrality and stability were evaluated through bootstrap analyses.

**Results:**

Both at T0 and T1, craving, impulsivity, and compulsive drug seeking formed a stable network, while the network structure dynamically reconstructed during abstinence. Drug craving and intention acted as the core node (highest centrality across abstinence stages). Longitudinal network analysis showed that resistance to thoughts and intentions strongly predicted subsequent impulsivity. The results highlight the dynamic interplay between craving and impulsivity in driving EUD symptoms over time.

**Conclusion:**

The findings provide insights into the temporal dynamics of EUD symptoms and suggest that craving and impulsivity play pivotal roles in the disorder’s progression. These results emphasize the need for targeted interventions that address these central symptoms to improve treatment outcomes.

## 1 Introduction

In recent years, etomidate, a traditional short-acting intravenous anesthetic, has emerged as a novel psychoactive substance (NPS) with non-medical abuse in China, South Korea, and other regions (1–4). The 2024 China Drug Situation Report and national drug surveillance data revealed it was the most frequently misused among narcotic and psychotropic drugs in China, with 86.4% of misusers younger than 35 (5). Per the UN Office on Drugs and Crime 2025 regional report, etomidate is rapidly spreading in East and Southeast Asia, with six new analogs identified in 2024 (6), underscoring its rising public health impact. Despite this, etomidate use disorder (EUD) has garnered relatively little scientific attention, as existing research on substance use disorders (SUDs) has focused primarily on more established drugs, leaving a gap in understanding the psychological, behavioral, and neurocognitive profiles of individuals with EUD.

Systematic investigations into the psychological and behavioral characteristics of individuals with EUD are still scarce. Most findings are derived from case reports or cross-sectional studies (7–9), which offer limited insights into the disorder’s symptom structure, evolution, and underlying neurobehavioral mechanisms. The Addictions Neuroclinical Assessment (ANA) model conceptualizes addiction via three major systems: reward/motivation enhancement, executive dysfunction, and negative affect (10). Similarly, both the Diagnostic and Statistical Manual of Mental Disorders, Fifth Edition (DSM-5) and International Classification of Diseases, 11th Revision (ICD-11) emphasize craving, compulsive drug-seeking, and impaired cognitive control as core features of SUDs (11, 12).

Craving is one of the central drivers behind the persistence and relapse of addictive behaviors (13). It is defined as a motivational state characterized by a desire to obtain a psychoactive substance or engage in an addictive behavior (14). Existing studies have found that craving can persist even after long-term abstinence (15). Further research suggests that craving fluctuates over time. For example, a study involving 153 individuals with methamphetamine use disorder found that craving intensity peaked during the 1–3 month abstinence period, showing an inverted “U”-shaped pattern (16). Another study on schizophrenia patients with SUDs indicated that craving levels typically peak during the acute withdrawal phase (1–4 weeks), followed by a rapid decline. By the sixth month, mean scores on the Visual Analog Scale (VAS) for craving had decreased to approximately 1.5 out of 10, reflecting a substantial and sustained reduction in craving (17). Closely related to craving, which refers to an individual’s inability to resist the urge to continue using drugs despite serious consequences. Compulsive drug seeking is a process of fundamental neural circuit remodeling that occurs over time and is typically described as a shift from goal-directed to habitual behavioral control. In the early stages of addiction, drug-seeking behavior is mainly driven by the positive reinforcing effects of the drug (i.e., pleasure); however, in the later stages of addiction, due to long-term drug exposure-induced neuroplasticity changes, drug-seeking behavior gradually becomes detached from reward expectations and evolves into an automatic behavior that persists without volitional control (18, 19). Cognitive control impairment is a key pathological mechanism mediating compulsive drug seeking. This regulatory system is responsible for top-down regulation that coordinates lower-level cognitive modules. Many studies have observed significant cognitive control deficits in individuals with addiction, particularly in inhibitory control and impulsivity (20–22). These three components—craving, compulsive drug seeking, and cognitive control impairment—may not exist in isolation and could potentially influence each other (23). Inhibitory control suppresses inappropriate responses through the prefrontal and subcortical circuits, while craving may be driven by the Go system in the prefrontal cortex, which encourages the formation of habits that enhance dopaminergic effects (24). Given their multidimensional nature, combining multiple assessment methods, such as self-report scales and behavioral tasks, is crucial for a comprehensive understanding of these phenomena.

Symptom network theory provides a novel perspective for deciphering the underlying structure of mental disorders. Distinct from traditional variable-centered approaches, network analysis eschews *a priori* assumptions about causal relationships among variables, instead uncovering direct symptom interactions, identifying key nodes, and clarifying intervention pathways (25). Research has shown that network analysis is a reliable and promising approach in the study of SUDs. For instance, a network analysis focusing on methamphetamine use disorder identified limited access to emotion regulation strategies, deficient emotional awareness, and non-acceptance of emotional responses as the nodes with the highest strength centrality (26). Another study applying network analysis to polysubstance users delineated specific substance-symptom combinations, highlighting drug overdose as a potent bridging symptom linked to a spectrum of substances and symptoms, which may reflect overall substance use severity (27). Additionally, a multicenter cross-sectional network analysis investigating gambling disorder (GD) among patients with methamphetamine use disorder (MUD) in Chinese drug rehabilitation centers revealed that gambling preoccupation and methamphetamine tolerance are core features, with the latter functioning as a bridging symptom between GD and MUD (28).

Nevertheless, most existing studies rely on cross-sectional data, precluding tracking symptom progression and identifying causal mechanisms. Longitudinal network analysis overcomes such limitations by capturing temporal symptom interactions, and research on other mental disorders, such as gaming disorder, depression, has confirmed the dynamic evolution of symptom structures over time (29–31). Its applications have also been documented in SUDs: for example, a panel network analysis of 1,829 adolescents found social motives, previous alcohol use, and openness predicted alcohol-related problems over time (32). Longitudinal network analysis of four waves of data from a large cluster-randomized controlled trial involving adolescents revealed that deliberate planning of binge drinking and uncontrollable intoxication states constitute core elements within the alcohol-related harms network (33). However, its application in EUD remains scarce.

The present study represents the first integration of cross-sectional network analysis and Cross-Lagged Panel Network (CLPN) modeling to examine both the static architecture and dynamic interplay among core symptoms (34)—such as drug craving, compulsive drug-seeking, and cognitive control deficits—in individuals with etomidate abuse. Assessments were conducted at admission and 1 month after short-term withdrawal, with the aim of identifying symptom nodes exhibiting temporal centrality and predictive utility for targeted intervention to reduce relapse rates. Aligned with ongoing policy cycles, including voluntary detoxification initiatives, the one-month observation window established here offers a feasible and structured framework to guide individualized rehabilitation pathways, optimize intervention timelines, and contribute to evidence-based policy formulation.

## 2 Methods

### 2.1 Participants

The study was conducted from June 2023 to December 2024 at a specialized drug rehabilitation center in Hunan Province, China. Individuals who were diagnosed with EUD based on the DSM-5 (35) and had used etomidate at least twice per month for a duration of 3 months or longer within the past year, with usage confirmed by hair toxicology testing, were eligible to participate in the study. Participants diagnosed with polydrug abuse, serious physical illness requiring ongoing medical treatment, or current or past major psychiatric disorders (e.g., schizophrenia, major depressive disorder, severe anxiety disorders, or bipolar disorder), were excluded from the study. This study was conducted in accordance with the Declaration of Helsinki and approved by the local research ethics committee. Written informed consent was obtained from all participants. For the 10-node network model, methodological guidelines mandate ≥ 20–30 participants per node (36, 37); accounting for potential follow-up attrition, a baseline sample size of at least 300 was needed.

### 2.2 Procedures and measures

All participants were interviewed face-to-face by psychiatrists trained in the field of addiction medicine. The survey consisted of five components: (1) sociodemographic information; (2) characteristics of etomidate use; (3) drug craving; (4) compulsive drug seeking; and (5) cognitive control ability (encompassing impulsivity and inhibitory control). The general demographic data of the subjects were collected, including age, years of education, marital state, employment status, personal income/month, whether they were only children, and family type. We collected information on the characteristics of etomidate use, including the age of initial use of etomidate, initial etomidate consumption dose, maximum single etomidate consumption dose and withdrawal duration. The baseline assessment (T0), conducted at the time of admission, included all five components listed above. A follow-up assessment was performed 1 month after admission (T1), covering components (3) to (5).

#### 2.2.1 Drug craving

The Drug Desire Questionnaire (DDQ) consists of 13 entries rated on a 7-point Likert scale (1 = not at all compliant, 7 = fully compliant) and divided into three dimensions: drug craving and intention, negative reinforcement craving, and drug control ability. Drug craving and intention assess the intensity of an individual’s desire to use drugs at the current moment and the planning involved in carrying out this behavior. Its characteristic is a direct reflection of the strength of motivation. Negative reinforcement craving assess the individual’s tendency to use drugs to escape pain, anxiety or solve problems in life. Its characteristic is to reflect the function of drugs as mood regulators. Drug control ability refers to an individual’s perceived ability to control their own drug use behavior and is characterized by diminished self-efficacy. Higher scores in the drug craving and intention and negative reinforcement craving dimensions indicate stronger craving, whereas higher scores in drug control reflect better self-control. This scale has demonstrated good reliability and validity in studies involving various addictive substances and multiple countries (with an average Cronbach’s coefficient of 0.86) (38).

#### 2.2.2 Compulsive drug seeking

The Obsessive Compulsive Drug Use Scale (OCDUS) is mainly used to assess an individual’s obsessive thoughts and behaviors related to drug use, to quantify the compulsive drug use behaviors of SUDs and their related psychological characteristics. It is categorized into three dimensions: craving thought intrusion, interference of drug, and resistance to thoughts and intention. Craving thought intrusion mainly measures the frequency of drug-related intrusive thoughts, and interference of drug assess the degree to which these craving thoughts interfere with daily life. Resistance to thoughts and intention evaluates an individual’s ability to resist drug temptation and whether there are specific intentions to relapse, reverse scoring. The OCDUS consists of 13 items and uses a 5-point Likert scale, where 1 indicates “not at all” and 5 indicates “very much.” A higher total score indicates more severe compulsive drug use behavior and higher craving in the subjects. This scale has been widely used in the research of various addictive substances and has good reliability and validity (Cronbach’s α = 0.87) (39, 40).

#### 2.2.3 Cognitive control ability

The Chinese version of the Barratt Impulsivity Scale, 11th edition (BIS-11), is a 30-item instrument designed to assess three distinct dimensions of impulsivity: cognitive impulsivity, motor impulsivity, and non-planning impulsivity. Cognitive impulsivity is characterized by difficulties in sustaining attention, heightened distractibility, and disorganized or rapid shifts in thought. Motor impulsivity involves restlessness, an inability to remain physically still, and the tendency to act without prior deliberation. Non-planning impulsivity is marked by the absence of clear future goals or structured planning, as well as a general lack of organization in daily functioning. It demonstrates good internal consistency, with a Cronbach’s α coefficient of 0.89, in populations with SUDs (41). Higher total scores reflect a greater tendency toward impulsive behavior.

The Stop Signal Task (SST) is a widely utilized cognitive control paradigm for assessing response inhibition, particularly within the context of substance addiction research (42). Reaction task: Press “F” with the left index finger for “O” in a green frame, and press “J” with the right index finger for “X” in a green frame. Stop task: Do not press any key when the letter’s frame turns red. Reaction trial process: First, a “+” appears in the center (100 ms), followed by the Go stimulus (1,200 ms), then the “correct/incorrect/slow” feedback (1,000 ms), and finally a “+” (400 ms). The Stop Signal Reaction Time (SSRT) reflects the latency with which an individual can inhibit an already initiated motor response. Elevated SSRT values indicate diminished inhibitory control (43). The task process is illustrated in Supplementary Figure S1.

### 2.3 Analytical approaches

#### 2.3.1 Descriptive analyses

All descriptive analyses were performed in R (version 4.1.1). Continuous variables are presented as median and interquartile range (IQR; 25–75%), and categorical variables as frequencies and percentages. The differences in clinical variables between the T0 and T1 time points were analyzed using the paired *t*-test if they met the assumption of normality; otherwise, the Wilcoxon signed-rank test was employed.

#### 2.3.2 Network estimations

Firstly, the “goldbricker” function in the “networktools” package (version 1.6.0) of R language was used to identify and eliminate redundant symptoms. The results indicated that no symptoms were excluded due to redundancy. Subsequently, the “qgraph” package (version 1.9.8) and the “bootnet” package (version 1.6) were employed to construct the network model (44). A Gaussian graphical model (GGM) based on partial correlation analysis was adopted to estimate the network structure. To reduce false associations and false positives, the graphical least absolute shrinkage and selection operator (GLASSO) was applied to regularize the network, thereby obtaining the most concise and conservative network model (45). Meanwhile, the extended Bayesian information criterion (EBIC) was utilized to select the best-fitting model (45). In the network, circles (nodes) represent individual symptoms, and the lines (edges) between nodes indicate the partial correlation association between two nodes after controlling for the influence of other symptoms. Blue edges represent positive partial correlations, while red edges represent negative partial correlations. The thickness of the edges reflects the absolute value of the partial correlation coefficient, with thicker edges indicating a stronger association.

#### 2.3.3 Centrality analysis

Node centrality is used to describe the connection degree of each node in a network and is a quantitative indicator to measure the importance and core of nodes in the network structure. It helps identify those nodes that occupy more core positions or have greater influence in the network (36). We drew on prior studies and adopted two indices: strength and expected influence (EI). Strength is the sum of connections to a focal node, calculated and visualized using the “centralityPlot” function in the “qgraph” package. EI outperforms earlier centrality indices in predicting node influence, so we calculated and reported it via the “networktools” package (46). This study thus focuses on strength and EI.

#### 2.3.4 Network accuracy and stability

This study employed the non-parametric bootstrap method (bootstrapped samples = 1,000) to assess the accuracy of strength, EI, edge weights in the network. By conducting 1,000 with-replacement samplings of the original dataset, the 95% confidence intervals of the strength, EI, edge weights obtained from each sampling were calculated to infer the distribution of the edge weights. If the 95% confidence intervals are narrow and have little overlap, it indicates that the estimation of the edge weights is relatively accurate (36). Stability is measured by calculating the correlation stability coefficient (CS-C) through the case -dropping bootstrap method (bootstrapped samples = 1,000). The CS-C should not be lower than 0.25, and it is better to be higher than 0.5. The accuracy and stability of the network are evaluated using the “bootnet” package (36).

#### 2.3.5 Cross-lagged panel network modeling

We employed the CLPN model to analyze longitudinal data, aiming to investigate the interrelationships among core symptoms of drug addiction over time. This model encompasses two types of effects: one is the influence of symptoms on themselves at different time points (autoregressive effect), and the other is the predictive effect of symptoms on other symptoms at different time points (cross-lagged effect) (47). To optimize the model, we processed the autoregressive and cross-lagged coefficients through LASSO regularization and selected the tuning parameters using 10-fold cross-validation (48). Additionally, we only reported the cross-lagged effects that remained significant after being filtered by a 0.1 threshold.

## 3 Results

### 3.1 Sample characteristics

A total of 361 participants in this study were included in the data analysis and completed the baseline assessment. The median age of participants was 21 years, the median years of education was 9 years, and the median of monthly personal income was 8,000 RMB. Among individuals with EUD, 52.3% were unmarried and single, 31.3% had no employment prior to admission, 25.7% came from single-parent families, and 77.3% were from families with multiple children. For EUD patients, the median days of abstinence for the EUD patients was10 days. The median age of first use of illegal psychoactive substances was 20 years. Regarding etomidate use, the initial drug dose was 0.5 g, and the drug maximum single dose was 1.68 g. Detailed sample characteristics are presented in Table 1.

**Table 1:** Basic information and clinical characteristics of EUD patients.

Variable	Baseline *n* (%)/Mdn (P25, P75)
Sample size	361
Age, year*	21 (18, 26)
Years of education, year*	9 (8, 11)
Marital state	
Never married	189 (52.3%)
Married or cohabiting	165 (45.7%)
Divorced or widowed	7 (2.0%)
Employment status	
Unemployed	113 (31.3%)
Employed	248 (68.7%)
Personal monthly income (Chinese Yuan, CNY)*	8,000 (5,000, 15,000)
Parental marital status	
Single parent family	93 (25.7%)
Intact family	268 (74.3%)
Sibling status	
Being an only child	82 (22.7%)
Having siblings	279 (77.3%)
Etomidate-related information	
Age of first onset, years*	20 (17, 25)
Initial etomidate consumption dose, grams*	0.50 (0.30, 1)
Maximum single etomidate consumption dose, grams*	1.68 (0.79, 3)
Withdrawal duration, days*	10 (8, 13)

*Data descriptive with median (interquartile range).

The 209 participants were followed up at 1 month. A total of 152 participants failed to complete the follow-up, with reasons including early release from rehabilitation facilities (38.3%), transfer to criminal detention (19.1%), voluntary withdrawal from the study (12.1%), and other administrative reasons (30.5%). Results showed significant improvements in multiple indicators at T1 compared to T0 (Table 2). Regarding compulsive drug seeking and cognitive control, all three dimensions of the OCDUS scale and the BIS scale exhibited downward trends, though these differences were not statistically significant. The SSRT declined significantly (264.22 ± 41.47 vs. 249.68 ± 31.68, *p* < 0.001). However, within the craving dimension, drug craving and intention significantly increased (*p* < 0.001). Similarly, negative reinforcement craving also significantly increased (*p* < 0.001), as were the scores of drug control ability (*p* < 0.001).

**Table 2:** Comparison of differences in clinical features between T0 and T1.

Variable	T0 (*n* = 209) M ± SD/Mdn (P25, P75)	T1 (*n* = 209) M ± SD/Mdn (P25, P75)	*t*/*Z*	*p*
D1	7.00 (3.00, 17.00)	13.00 (9.00, 20.00)	−5.343^a^	<0.001
D2	5.00 (3.00, 12.00)	11.00 (7.00, 16.00)	−6.630^a^	<0.001
D3	5.00 (4.00, 8.00)	7.00 (5.00, 10.00)	−3.573^a^	<0.001
O1	12.30 ± 4.73	11.05 ± 4.02	3.349^b^	0.001
O2	10.22 ± 4.70	9.72 ± 4.41	1.363^b^	0.174
O3	5.82 ± 1.90	5.80 ± 2.03	0.131^b^	0.896
B1	29.81 ± 6.30	28.90 ± 6.63	1.356^b^	0.177
B2	25.86 ± 7.85	24.81 ± 6.93	1.512^b^	0.132
B3	28.68 ± 7.64	27.59 ± 7.61	1.351^b^	0.178
SSRT	264.22 ± 41.47	249.68 ± 31.68	4.210^b^	<0.001

For indicators that did not conform to the normal distribution (D1, D2, D3), the statistical results are presented as median (25th percentile, 75th percentile) [Mdn (P25, P75)], and the Wilcoxon signed-rank test (denoted as “a”) was applied for the comparison between T0 and T1. For indicators that conformed to the normal distribution, the statistical results are presented as mean ± standard deviation (M ± SD), and the paired *t*-test (denoted as “b”) was used for the comparison between T0 and T1.

### 3.2 Cross-sectional symptom networks

#### 3.2.1 Connections among symptoms

**Figure 1:**
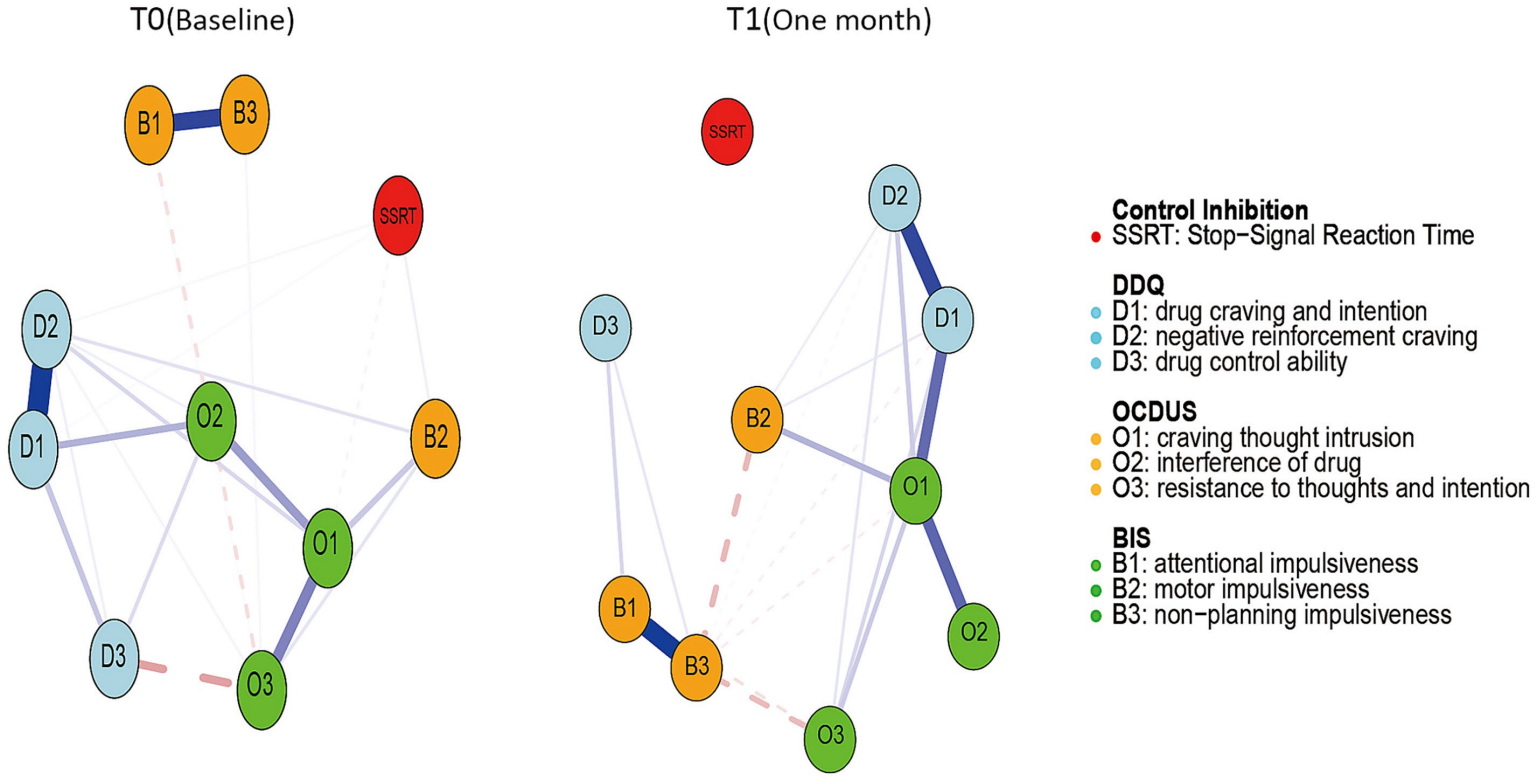
The network structure of symptoms at two time points. Nodes represent 10 core symptoms, and the lines between nodes indicate the strength of the correlation. The thicker the line, the stronger the connection. Blue indicates a positive correlation, and red indicates a negative correlation.

The core symptom networks at baseline (T0), 1 month (T1), are shown in Figure 1. We mostly discussed connections with edge strengths greater than 0.30, indicating relationships that are moderate to strong. At T0, we found six strong connections, namely, edges between attentional impulsiveness and non-planning impulsiveness (B1: B3), drug craving and intention and negative reinforcement craving (D1: D2), and craving thought intrusion and interference of drug (O1: O2), craving thought intrusion and resistance to thoughts and intention (O1: O3), drug craving and intention and interference of drug (D1: O2), and drug control ability and resistance to thoughts and intention (D3: O3) with partial correlation coefficients between 0.37 and 0.9. At T1, we found that the overall network connections became sparse following 1 month of follow-up, but the key edges that were strong at T0 remained robust. There were five edges that showed strong connections, with coefficients ranging from 0.37 to 0.76. In addition to the three edges we found at T0, the connection between drug craving and intention and craving thought intrusion (D1: O1), motor impulsiveness and craving thought intrusion (B2: O1) was enhanced. In general, several strongly connected edges were observed, such as B1: B3, D1: D2 and O1: O2, which were consistent across two networks. Correspondingly, some specific connections describe the differences between the two networks. For example, the connections between O1: O3, D1: O2, and D3: O3 were initially strong, but weakened after 1 month. In contrast, the connection between D1: O1 and B2: O1, which was weak at T0, strengthened at time point T1.

#### 3.2.2 Centrality analysis

**Figure 2:**
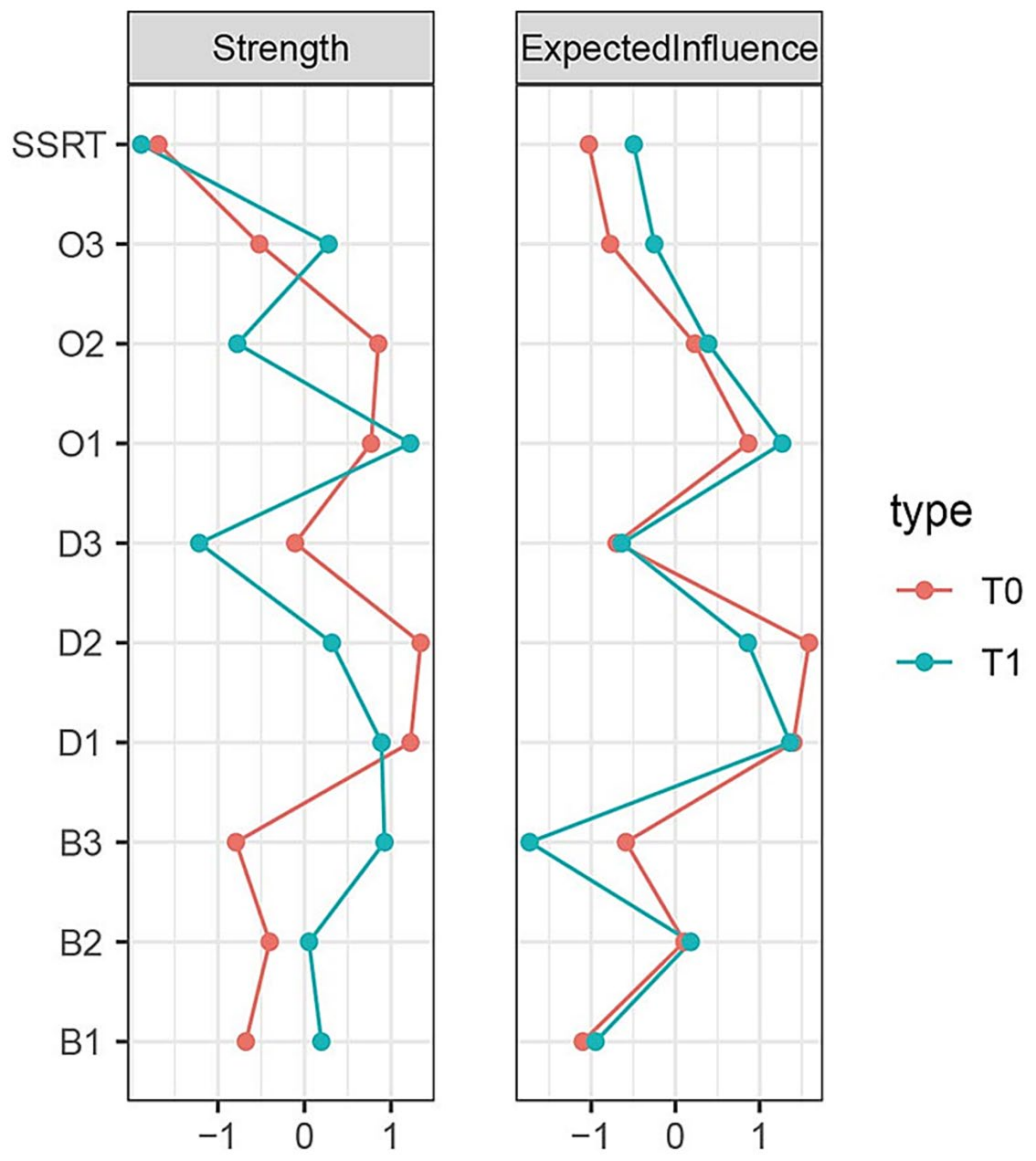
The standardized centrality index of each node in the symptom network at two time points. Intensity represents the sum of the absolute values of the weights of the edges to a specific node, while EI refers to the sum of the positive and negative weights connected to a node. The red and green lines represent the two time points of T0, T1.

The strength and EI centrality of each time point in the network are shown in Figure 2. According to the results, at T0, the symptoms related to D1, D2, O2 exhibited higher centrality estimates. At T1, D1, and O1 had high centrality estimates. SSRT and D3 were the lower centrality nodes across all two visit points. Overall, as the abstinence period lengthens, symptoms exhibiting high strength and expected influence became more prominent, while nodes with low centrality showed a decreasing trend. D1 plays an important role in the network at both time points.

#### 3.2.3 Network accuracy and stability

**Figure 3:**
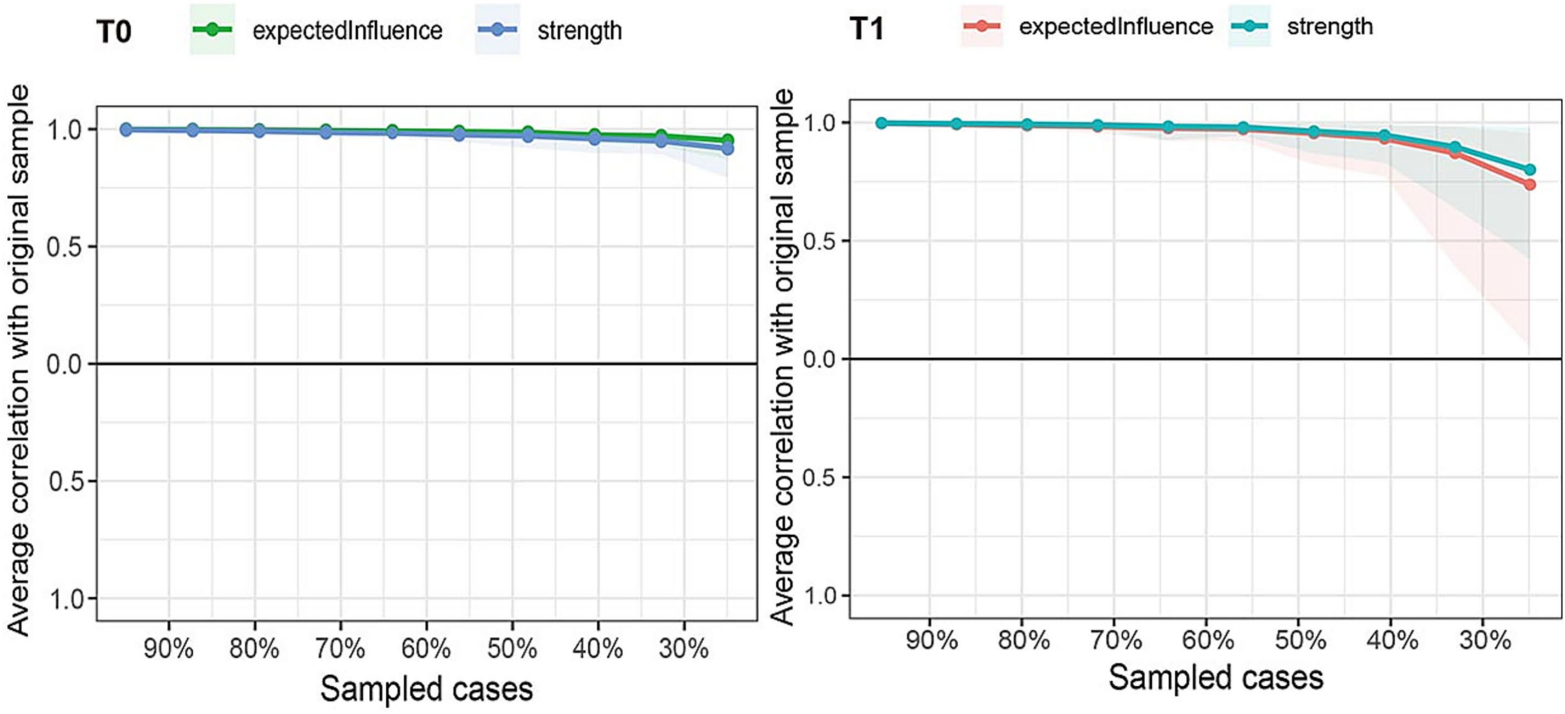
Two subplots illustrate network stability analysis, correlation stability coefficient (CS-coefficient) values indicate greater network stability.

Both the network strength and EI values demonstrated good stability. At the T0 period, the CS-C value was 0.75, indicating that even after removing 75% of the samples, the node indicators of the network remained stable without significant changes. At the T1 period, the CS-C value was 0.67 (Figure 3). The 95% confidence interval of the edge weights calculated by the Bootstrap method was relatively narrow, and the original edge weights were basically consistent with the average value of the edge weights calculated by the Bootstrap method, suggesting that the edge weights were stable and accurate (Supplementary Figures S4C, S5C).

### 3.3 CLPN analysis

**Figure 4:**
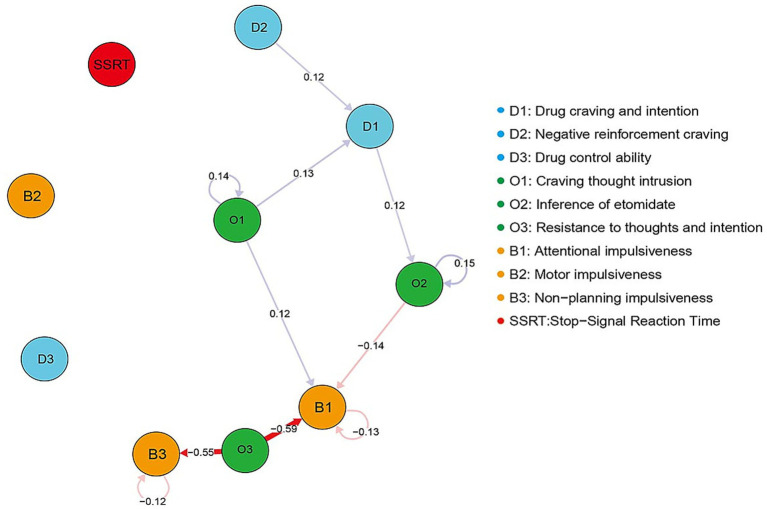
Cross-lagged panel network of core symptoms. Nodes indicate symptoms in the network, and edges indicate longitudinal associations between nodes. Blue lines indicate positive associations, and red lines indicate negative associations.

The core clinical symptoms of EUD were plotted as a directed CLPN, as shown in Figure 4, with arrows relaying temporal associations of the edges within and across constructs. Only substantial edges (edge values greater than 0.1) are displayed in the network to make the graph more intuitively interpretable. Our findings indicate that baseline clinical symptoms exert a predictive effect on the following assessments. Specifically, Baseline symptoms D2 (negative reinforcement craving) and O1 (craving thought intrusion) was a significant predictor of D1 (drug craving and intention) at T1 (1 month later). Furthermore, D1 at T0 positive predictive effect on O2 at T1 (interference of drug), as did O1 at T0 versus B1 (attentional impulsiveness) at T1. Baseline symptom O3 (resistance to thoughts and intentions) negatively predicted B1 (attentional impulsiveness) and B3 (non-planning impulsiveness) at T1. Similarly, baseline O2 also showed negative predictive utility for B1 at T1. The nodes with the autoregressive coefficients are O1, O2, B1, and B3. In-prediction and out-prediction of CLPN are presented in Supplementary Figure S2. O3 (resistance to thoughts and intentions) exhibits the highest out-EI, which is significantly greater than that of other symptoms in the network. It tends to have a greater influence on other variables in the time network. In terms of in-EI estimates, B1 (attentional impulsiveness) shows the highest value, suggesting that the symptom are strongly predicted by others. Supplementary Figure S3 shows autoregressive edges. The strongest autoregressive path is O2 (interference of drug), especially within T0-T1.

## 4 Discussion

### 4.1 Main findings

This study employed a network analysis approach to explore the dynamic changes in the symptom network of individuals with EUD during a one-month short-term withdrawal period. The findings indicated that some symptom associations remained relatively stable, such as attentional impulsiveness/non-planning impulsiveness (B1: B3), drug craving/intention and negative reinforcement craving (D1: D2), and craving thought intrusion/interference of drug (O1: O2). And drug craving and intention (D1) occupied a relatively central position in the network. CLPN analysis further revealed that all compulsive drug-seeking symptoms, including O1, O2, and resistance to thoughts and intentions (O3), may predict changes in drug craving and impulsivity symptoms.

### 4.2 Cross-sectional network

Cross-sectional analyses across two time points reveal that many positive connections within the network remain stable over the study period. Consistent with prior SUDs research, these stable links support framing impulsivity, craving, and compulsive drug-seeking as distinct yet multidimensional constructs (49–52). There are strong network connections between various dimensions of these three core SUD symptom clusters. Focusing specifically on drug craving, the persistent connection between drug craving/intention and negative reinforcement craving points to a dual-drive mechanism underlying EUD-related craving: it arises both from anticipation of the drug’s positive rewarding effects (positive reinforcement) and the urgent drive to escape aversive emotional states (negative reinforcement). Notably, this dual motivational pattern remains robust across time points, highlighting the enduring nature of craving experiences in EUD. Previous research within the ANA framework clearly indicates that individuals with addiction exhibit enhanced negative emotional responses to various stimuli, accompanied by overall self-reported dysphoric mood states (10). This negative emotional state is highly positively correlated with increased craving for drugs of abuse and relapse risk (53, 54). In states of intense craving, an individual’s capacity for cognitive control is significantly impaired, and intrusive thoughts become more frequent and increasingly difficult to suppress. This progression often culminates in compulsive drug use. Turning to impulsivity in EUD, distinct subdimensions play divergent key roles: cognitive impulsivity contributes critically to both initial drug initiation and relapse, while non-planning impulsivity emerges as a primary driver of sustained abstinence failure and social functional impairment (55).

The cross-sectional results also suggest that the structure of EUD symptom network changes with prolonged abstinence time. The association strength between drug craving and intention and craving thought intrusion (D1: O1) increased over time. In contrast, the association between drug craving and intention and interference of drug (D1: O2) was strong at baseline but gradually weakened after 1 month. These changes indicate the network structure is not static but shows significant dynamic reconstruction patterns as abstinence time extends. Compared to methamphetamine use disorder, where craving peaks at 3 months (16), EUD’s network restructuring within 1 month suggests a faster symptom evolution, possibly due to etomidate’s distinct pharmacological profile.

This study also found that drug craving and intention (D1) consistently emerged as the most central symptom in EUD patients across both cross-sectional time points. This symptom showed extremely high strength centrality values in both wave 1 and wave 2 assessments, indicating that it maintains the closest and most extensive associations with other symptom nodes in the network. This finding is highly consistent with extensive empirical research in the field of SUDs. Previous studies on various addictive substances such as alcohol, cocaine, and opioids have all identified craving as a core symptom and one of the diagnostic criteria for SUDs (56, 57). Our research results further extend this understanding, demonstrating that in EUD involving this novel psychoactive substance, drug craving similarly occupies a central position in the symptom network.

### 4.3 Cross-lagged panel network

The CLPN analysis revealed complex temporal relationships among EUD symptoms. Baseline negative reinforcement craving (D2) positively predicted drug craving and intention (D1) at 1 month, a finding that supports the negative reinforcement theoretical model of addiction (58, 59). This predictive relationship indicates that individuals’ drug use motivation to alleviate negative emotional states gradually evolves into more direct and intense drug craving experiences over time. The negative prediction of interference of drug (O2) on cognitive impulsivity (B1), the positive prediction of craving thought intrusion (O1) on cognitive impulsivity (B1), and the negative predictions of resistance to thoughts and intention (O3) on both B1 and B3 suggest the possibility that different types of cognitive symptoms may influence impulse control through different mechanisms (60, 61): certain cognitive processes may potentially exacerbate impulsivity (such as O1 → B1), while others might serve a protective function (such as the negative relationships of O3 → B1/B3). Resistance to thoughts and intention (O3) showed the highest out-EI value in EUD individuals, indicating that it may play a central role in activating and predicting the occurrence of other symptoms. It could potentially represent a metacognitive control ability, namely the individual’s capacity to identify and resist negative cognitive content (62). Its high out-expected influence suggests that the strength of this cognitive control ability may have extensive effects on the future state of the entire symptom network. This finding provides preliminary support for the potential applicability of intervention strategies based on mindfulness and metacognitive training, though further research is needed to establish these relationships.

### 4.4 Limitations

Although our study provides valuable insights into the clinical core symptom networks of individuals with EUD, several limitations should be acknowledged. First, the 42.1% one-month dropout rate was primarily due to external factors (early release, administrative transfers), which reduces systematic bias concerns but cannot completely rule out unmeasured characteristics affecting results. Second, the study did not account for other factors—including negative affect, depression, and anxiety. Future research employing larger, more diverse samples should incorporate these variables to develop a more comprehensive clinical model of EUD. Third, participants were recruited from a single center and were predominantly male, which may limit the generalizability of the findings. Therefore, future multi-center studies with gender-balanced samples are essential to validate and extend the current results.

## 5 Conclusion

This study is the first to systematically apply network analysis methods to reveal key characteristics of symptom networks in individuals with EUD: drug craving and intention serves as the core node of the symptom network, consistently showing the highest centrality across different abstinence periods; the symptom network structure undergoes dynamic reconstruction over time during abstinence; longitudinal analysis reveals that resistance to thoughts and intentions exerts a strong predictive influence. These findings provide a network-theoretical foundation for the treatment of EUD, suggesting that interventions should prioritize targeting core symptoms and high-influence nodes.

## Data Availability

The original contributions presented in the study are included in the article/Supplementary material, further inquiries can be directed to the corresponding author.
