# Supplementary material for: Dynamic symptom networks in etomidate use disorder: a cross-lagged panel network analysis of craving, compulsive drug seeking, and cognitive control
Source: Front Public Health. 2026 Jan 20;13:1725123. doi: 10.3389/fpubh.2025.1725123 (PMC12864409; doi:10.3389/fpubh.2025.1725123)
Supplement: Supplementary file 1 [file Data_Sheet_1.pdf]

The Stop Signal Task (SST)

The Stop-Signal Task (SST) is a classic behavioral paradigm designed based on the theory of response inhibition(Luijten et al., 2014). In this task, participants are required to quickly and accurately complete either a go task or a stop task. The prefrontal control and inhibitory functions are quantified by tracking indicators such as the successful inhibition rate and stop-signal reaction time.

The SST in this study consists of 60% go tasks and 40% stop tasks, with the two types of tasks presented randomly. For the go task, when a green-framed "O" appears on the screen, participants press the "F" key with their left index finger; when a green-framed "X" appears, they press the "J" key with their right index finger. For the stop task, when the frame around the letter changes from green to red, participants need to inhibit the urge to press a key and refrain from responding. In go trials, first, a "+" serving as a fixation point is presented at the center of the screen for 100 ms, followed by the presentation of the Go stimulus for 1200 ms. Then, feedback of "correct/incorrect/slow" is displayed for 1000 ms, and finally, a "+" is shown for 400 ms. The red frame acts as the stop signal, and the time interval from the green frame to the red frame is referred to as the stop-signal delay (SSD). The study employs a parameter adaptive tracking method to automatically adjust the duration of SSD to accommodate participants with different reaction speeds. The SSD starts at 250 ms and is dynamically adjusted by the program (with SSD increasing by 50 ms for successful inhibition and decreasing by 50 ms for failed inhibition, within a range of 0-1200 ms). The task flow is shown in *Figure* S1.

This study adopts Go reaction time, stop-signal delay (SSD), and stop-signal reaction time (SSRT) as the main measurement indicators. SSRT refers to the time from the appearance of the stop signal to the participant's completion of the stop task, that is, the internal reaction time for successfully inhibiting a stop task. A shorter SSRT indicates stronger control and inhibition ability in participants(Jansen, 2023).

Prior to the formal experiment, participants are required to undergo practice. The practice consists of two parts: the first part includes 30 trials, all of which are go tasks where the green frame does not turn red, and participants only need to press the correct key as quickly as possible; the second part comprises 20 trials, including go tasks (60%) and stop tasks (40%). The formal experiment consists of 240 trials (80 trials/block * 3 blocks = 240 trials). After completing each block, participants can close their eyes to rest and then press "R" to continue. The reaction times in the formal experiment must not be slower than 1.25 times the average reaction time during practic

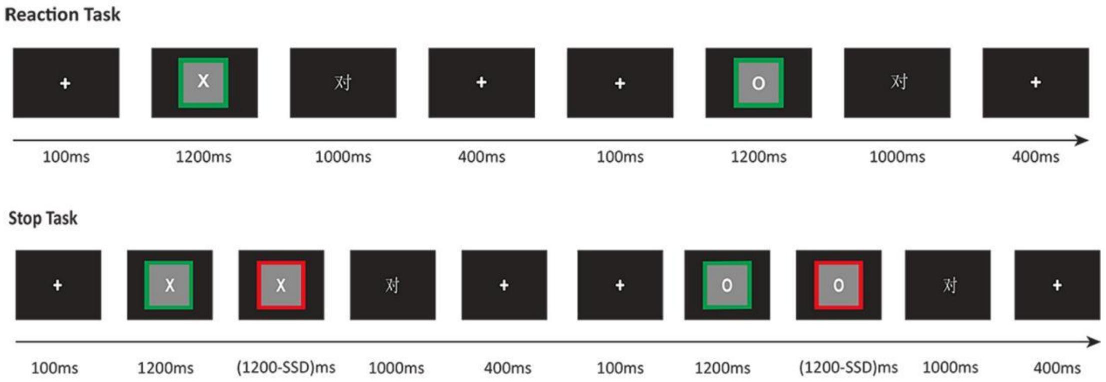

Figure S1 Stop Signal Task Experiment Flow

Reference

Jansen, J. M. (2023). Mediating effects of impulsivity and alexithymia in the association between traumatic brain injury and aggression in incarcerated males. *Aggressive Behavior, 49*(6), 629-642. https://doi.org/10.1002/ab.22101

Luijten, M., Machielsen, M. W., Veltman, D. J., Hester, R., de Haan, L., & Franken, I. H. (2014, May). Systematic review of ERP and fMRI studies investigating inhibitory control and error processing in people with substance dependence and behavioural addictions. *J Psychiatry Neurosci, 39*(3), 149-169. https://doi.org/10.1503/jpn.130052

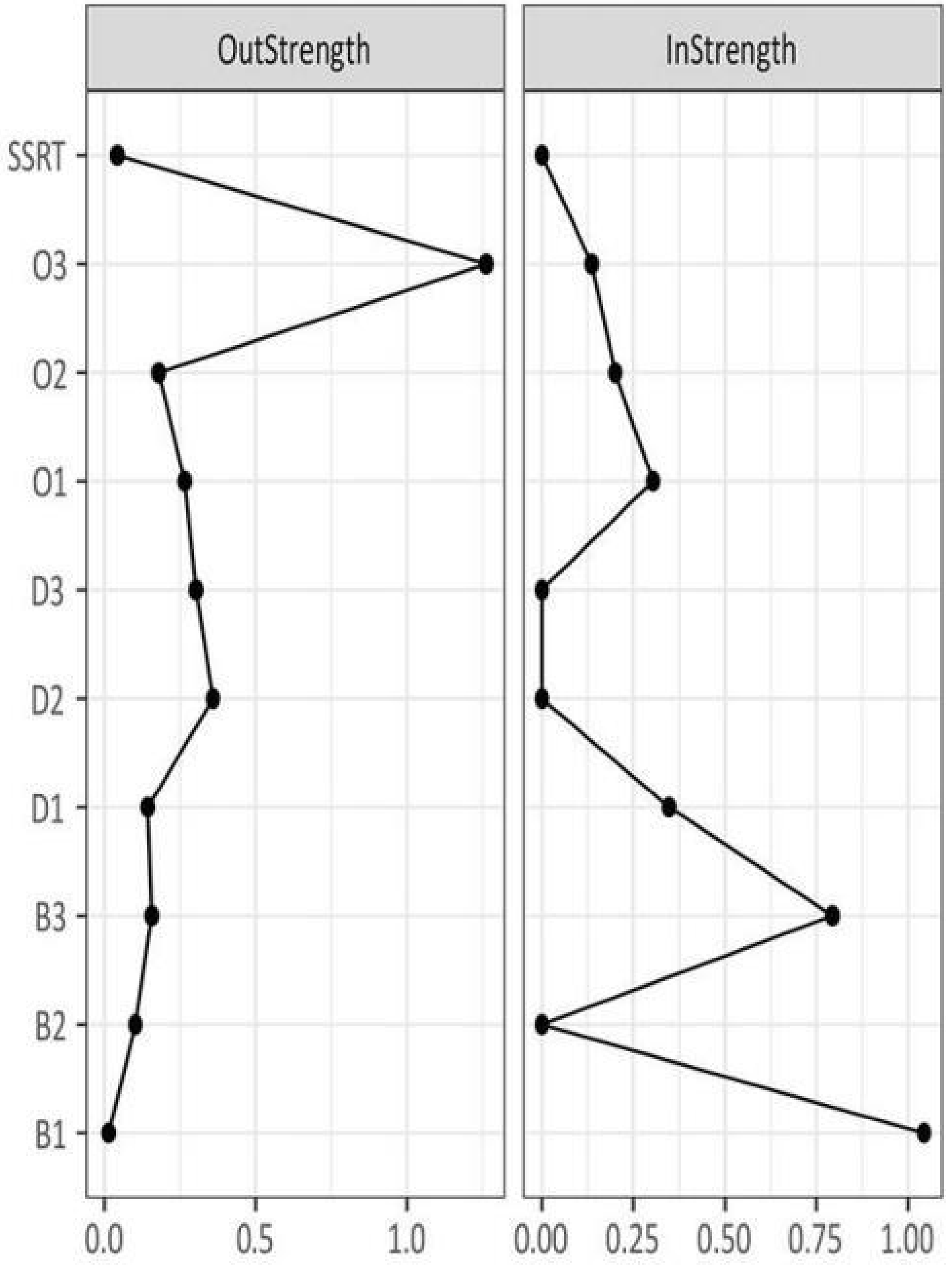

Figure S2. In-prediction and out-prediction of Cross-lagged panel network.

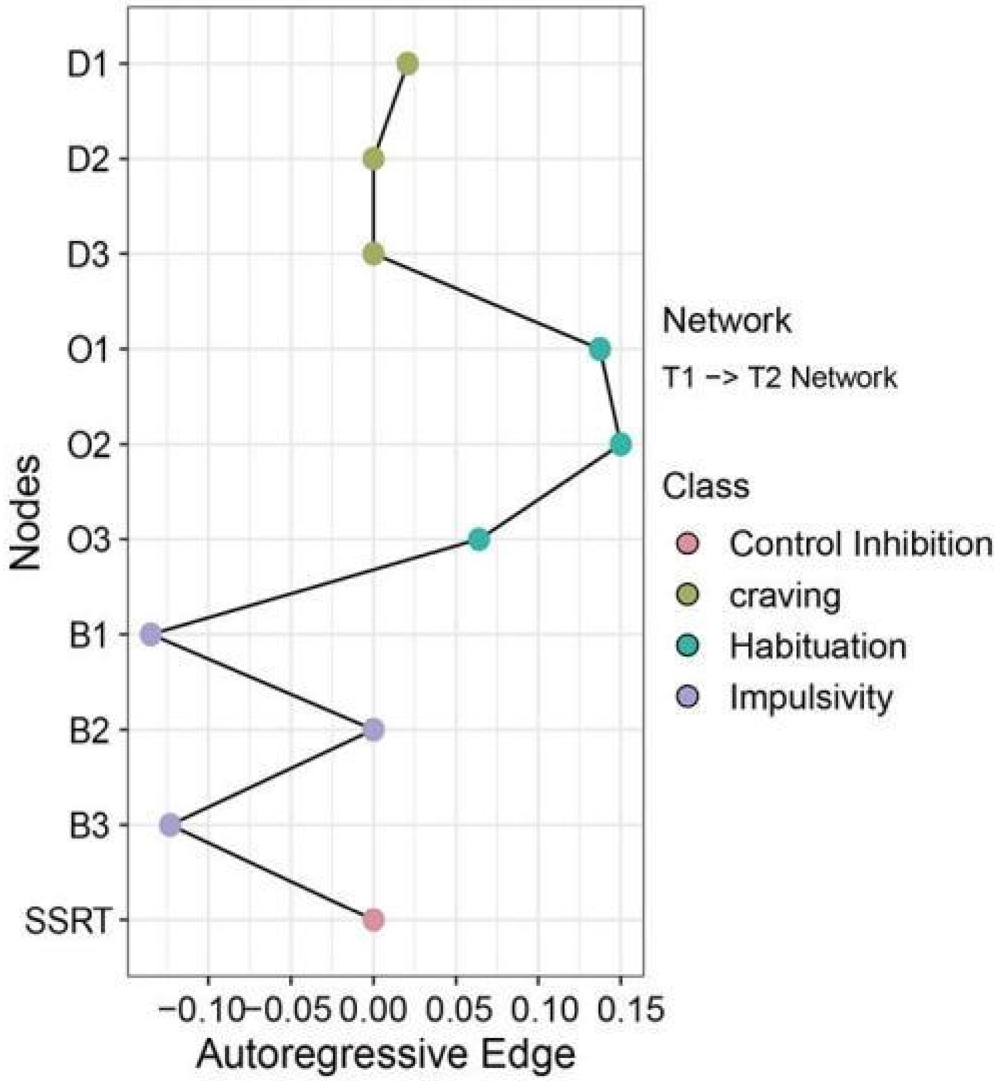

Figure S3. Autoregressive edges.

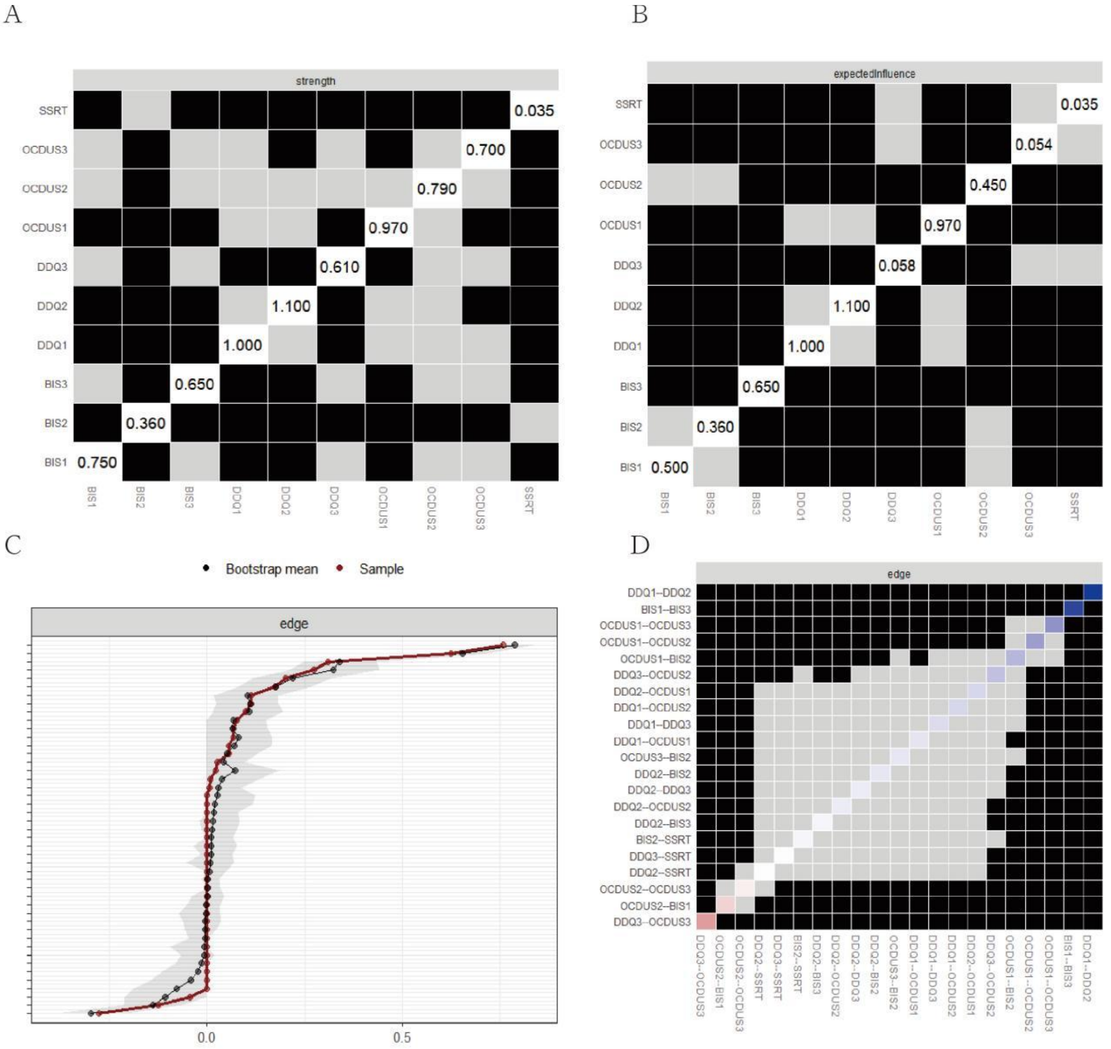

Figure S4. Validation of the accuracy of the T0 network

A. Test of differences in strength among nodes of the T0 network model B. Test of differences in expected influence among nodes of the T0 network model C. Confidence interval plot for validating the precision of estimated edge weights in the T0 network model D. Test of differences among edges of the T0 network model

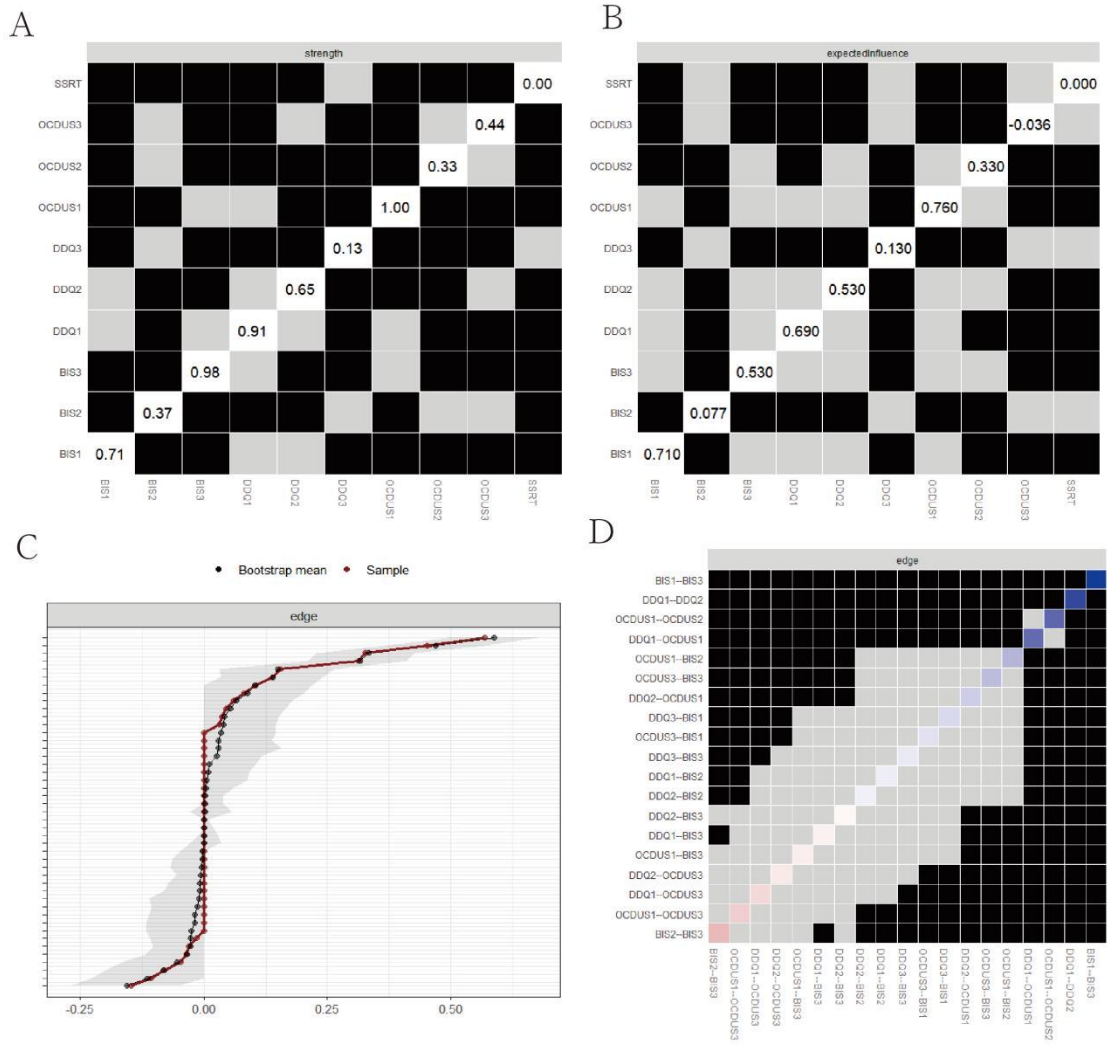

Figure S5. Validation of the accuracy of the T1 network

A. Test of differences in strength among nodes of the T1 network model B. Test of differences in expected influence among nodes of the T1 network model C. Confidence interval plot for validating the precision of estimated edge weights in the T1 network model D. Test of differences among edges of the T1 network model

Basic Information

**Evaluator: ________**	**Evaluation Center:**
Admission Date: _____ Example: 20201125	Name:
Contact Phone (Self or Family):	
Gender: □Male □Female Date of Birth: ________ Example: 20201125<br>Age: _____ years	
Ethnicity: □Han=1; □Hui=2; □Mongolian=3; □Others=4 (Please specify:)	
Education Level: □1=Primary school or below; □2=Junior high school; □3=Senior high school/Technical secondary school/Technical school; □4=Junior college; □5=Undergraduate; □6=Postgraduate or above;	
Years of Education: □□ years	
Employment Status Before Admission: □Unemployed=1; □Part-time job=2; □Full-time job or student=3; □Freelance=4;	
Occupation: □1=Unemployed; □2=Retired/Resigned; □3=Student; □4=Doctor; □5=Teacher; □6=Civil Servant; □7=Engineer;□8=Service industry; □9=Self-employed; □10=Freelance; □11=Worker; □12=Farmer; □13=Soldier; □14=Transportation industry; □15=Professional and technical personnel; □16=Researcher; □17=Staff of enterprises and institutions; □18=Employee in entertainment venues;<br>□19=Other______	
Personal income, month———	
Parental Marital Status □Single parent family □Intact family	
Sibling Status □Being an only child □Having siblings	
Marital Status: □Unmarried=1; □Married=2; □Divorced=3; □Widowed=4; □Cohabiting=5;	
**Current Comorbid Physical Diseases:**	
Diabetes: □Yes □No	Hypertension: □Yes □No
Craniocerebral Injury: □Yes □No	Epilepsy: □Yes □No
Periodic Paralysis: □Yes □No	Cerebrovascular Disease: □Yes □No
Other metabolic and endocrine diseases: (such as hypoglycemia, dyslipidemia, obesity, gout, osteoporosis, hypokalemia, hyperkalemia, hyponatremia, hypernatremia, and other water, electrolyte and acid-base balance disorders, etc.)<br>□Yes □No	

Etomidate-related information

Age of first onset, years	
Initial etomidate consumption dose, grams	
Maximum single etomidate consumption dose, grams	
Withdrawal duration, days	

Severity of Substance Use Disorder (DSM-5)

Please recall the past 12 - month period (for those in withdrawal, please recall the 12 - month period before treatment). If you have any of the following situations, please check the box if applicable:

1. The intake of the substance is often in larger amounts or over a longer period than intended;
2. There is a persistent desire to cut down or control substance use, or there have been unsuccessful attempts to do so;
3. A great deal of time is spent in activities related to obtaining the substance, using the substance, or recovering from its effects;
4. There is a craving or intense desire or urge to use the substance;
5. Recurrent substance use results in failure to fulfill major role obligations at work, school, or home;
6. Continued substance use despite having persistent or recurrent social or interpersonal problems caused or exacerbated by the substance use;
7. Important social, occupational, or recreational activities are given up or reduced because of substance use;
8. Recurrent substance use in situations in which it is physically hazardous;
9. Continued substance use despite knowledge of having a persistent or recurrent physical or psychological problem that is likely to have been caused or exacerbated by the substance;
10. Tolerance, defined as either of the following:
a. A need for markedly increased amounts of the substance to achieve intoxication or the desired effect;
b. A markedly diminished effect with continued use of the same amount of the substance;
11. Withdrawal, manifested by either of the following:
a. The characteristic withdrawal syndrome for the substance occurs after cessation or reduction of use (as follows):
Methamphetamine withdrawal: After situation a, feelings of dysphoria and irritability occur within hours to days, along with at least 2 of the following symptoms: fatigue; vivid, unpleasant dreams; insomnia or hypersomnia; increased appetite; psychomotor retardation or agitation.
Opioid withdrawal: After situation a, 3 or more of the following symptoms occur, lasting from minutes to days: dysphoric mood; nausea or vomiting; muscle aches; lacrimation or rhinorrhea; pupillary dilation; piloerection or sweating; diarrhea; yawning; fever; insomnia.
b. The substance is used to relieve or avoid withdrawal symptoms;
